# Retraction: Mitochondrial fission and mitophagy reciprocally orchestrate cardiac fibroblasts activation

**DOI:** 10.3389/fcell.2022.1020548

**Published:** 2022-08-24

Following publication, concerns were raised regarding the integrity of the images in the published figures. The authors failed to provide a satisfactory explanation during the investigation, which was conducted in accordance with Frontiers’ policies.

Given the concerns, the editors no longer have confidence in the findings presented in the article.

The authors do not agree to this retraction.

This retraction was approved by the Chief Editors of Frontiers in Cell and Developmental Biology and the Chief Executive Editor of Frontiers.

